# An account of the taxonomy and distribution of Syllidae (Annelida, Polychaetes) in the eastern Mediterranean, with notes on the genus *Prosphaerosyllis* San Martín, 1984 in the Mediterranean

**DOI:** 10.3897/zookeys.150.2146

**Published:** 2011-11-28

**Authors:** Sarah Faulwetter, Georgios Chatzigeorgiou, Bella S. Galil, Christos Arvanitidis

**Affiliations:** 1 Department of Zoology – Marine Biology, Faculty of Biology, National and Kapodestrian University of Athens, Panepistimiopolis, 15784, Athens, Greece; 2 Department of Biology, University of Crete, 71409 Heraklion, Crete, Greece; 3 National Institute of Oceanography, Israel Oceanographic and Limnological Research, POB 8030, Haifa 31080, Israel; 4 Institute of Marine Biology and Genetics, Hellenic Centre for Marine Research, 71003 Heraklion, Crete, Greece

**Keywords:** Polychaetes, Syllidae, eastern Mediterranean Sea, taxonomy, distribution, new records, alien species

## Abstract

The syllid fauna of three locations in Crete and Israel (eastern Mediterranean Sea) was studied, yielding 82 syllid species, many of which were found for the first time in the respective areas: Seventeen species were recorded for the first time on the Israeli coasts and 20 in Greek waters. *Perkinsyllis augeneri* (Hartmann-Schröder, 1979) and *Prosphaerosyllis chauseyensis* Olivier et al., 2011 are new records for the Mediterranean Sea. Detailed information is given on the morphology, ecology and distribution of the species recorded for the first time in the studied areas. In addition, an update on the distribution of the genus *Prosphaerosyllis* San Martín, 1984 in the Mediterranean is given and an identification key to the Mediterranean species is provided.

## Introduction

The Syllidae are a highly diverse family of polychaetes with currently around 900 valid species belonging to over 80 genera (pers. obs.) and have recently received considerable taxonomic and phylogenetic research effort, including a high number of new taxon descriptions (e.g. Aguado et al. 2007, Aguado and San Martín 2009, De Matos Nogueira et al. 2001, San Martín 2005, 2008, San Martín and Hutchings 2006, San Martín et al. 2009). Syllids are (usually) small-sized polychaetes with a high diversity of morphological and ecological features and are found globally on all types of substrates from the intertidal to the abyss (San Martín 2003).

**Figure 1.**
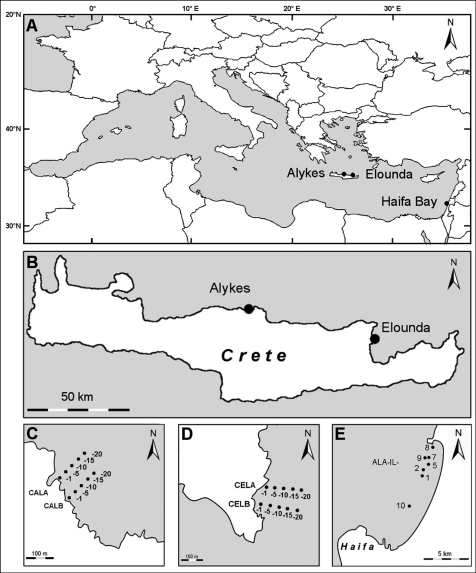
Map of the sampling stations **A** Location of the stations in the Mediterranean **B** Locations of the two sampling stations in Crete **C** Alykes **D** Elounda **E** Haifa Bay.

The present study contributes to the current knowledge of the syllid fauna of three different locations in the eastern Mediterranean Sea: two in Crete, one in Israel. The material has been collected in the framework of two different research programmes and from two different habitats (Fig. 1, Table 1): a) hard-bottom samples from Crete have been obtained within the NaGISA project (Natural Geography in Shore Areas, http://www.nagisa.coml.org), a field project of the Census of Marine Life (COML, http://www.coml.org); b) soft-sediment samples from the Israeli coast have been obtained in the framework of a project focusing on the soft bottom benthos of Haifa Bay. In all samples, Syllidae were highly abundant and yielded many species recorded for the first time in the respective area, as well as a species new to science (Faulwetter et al. 2011).

**Table 1.** Sampling stations and their characteristics

**Station Code**	**Location**	**Coordinates**	**Depth**	**Habitat**
ALA-IL-1	Haifa Bay, Israel	32°53.792'N, 35°03.928'E	13.1 m	Fine to medium sand
ALA-IL-2	Haifa Bay, Israel	32°54.052'N, 35°03.905'E	13.9 m	Sand of mixed grain sizes
ALA-IL-5	Haifa Bay, Israel	32°54.259'N, 35°04.160'E	11.4 m	Silty sand
ALA-IL-7	Haifa Bay, Israel	32°54.544'N, 35°04.093'E	10.5 m	Sand of mixed grain sizes with silt
ALA-IL-8	Haifa Bay, Israel	32°55'N, 35°04.239'E	7.8 m	Coarse sand with silt
ALA-IL-9	Haifa Bay, Israel	32°54.518'N, 35°03.950'E	8.7 m	Coarse sand
ALA-IL-10	Haifa Bay, Israel	32°52.509'N, 35°03.520'E	12.8 m	Medium to coarse sand
CALA-1, CALB-1	Alykes, Crete, Greece	35°24.95'N, 24°59.25'E	1 m	*Cystoseira* spp.*, Fucus virsoides*
CALA-5, CALB-5	Alykes, Crete, Greece	35°24.95'N, 24°59.25'E	5 m	Filamentous Chlorophyceae, *Amphiroa* sp., *Padina pavonica*
CALA-10, CALB-10	Alykes, Crete, Greece	35°24.95'N, 24°59.25'E	10 m	*Cystoseira* spp., filamentous Chlorophyceae
CALA-15, CALB-15	Alykes, Crete, Greece	35°24.95'N, 24°59.25'E	15 m	Filamentous Chlorophyceae, filamentous Phaeophyceae
CALA-20, CALB-20	Alykes, Crete, Greece	35°24.95'N, 24°59.25'E	20 m	Filamentous Phaeophyceae, *Bryopsis* sp., *Caulerpa* spp.
CELA-1, CELB-1	Elounda, Crete, Greece	35°15.1'N, 25°45.5'E	1 m	*Jania* sp., *Dasycladus clavaeformis*, Porifera spp., *Litophyllum* sp.
CELA-5, CELB-5	Elounda, Crete, Greece	35°15.1'N, 25°45.5'E	5 m	*Jania* sp., *Dasycladus clavaeformis, Litophyllum* sp., *Amphiroa* sp.
CELA-10, CELB-10	Elounda, Crete, Greece	35°15.1'N, 25°45.5'E	10 m	Filamentous Phaeophyceae, *Jania* sp., Porifera spp., *Bryopsis* sp.
CELA-15, CELB-15	Elounda, Crete, Greece	35°15.1'N, 25°45.5'E	15 m	Filamentous Phaeophyceae, *Jania* sp., *Peyssonellia* sp., filamentous Chlorophyceae
CELA-20, CELB-20	Elounda, Crete, Greece	35°15.1'N, 25°45.5'E	20 m	*Padina pavonica*, filamentous Chlorophyceae, *Amphiroa* sp.

In the Mediterranean Sea, syllids have been studied by numerous authors in extensive taxonomic and biogeographic works (e.g. Ben-Eliahu 1977a, 1977b, Campoy 1982, Çinar 1999, San Martín 1984b, 2003, Musco and Giangrande 2005), however, most research on the taxon is being carried out in the western Mediterranean basin, whereas the syllid fauna of the eastern Mediterranean has only recently started to be investigated more intensely (e.g. Ben-Eliahu 1977a, 1977b, Çinar 1999, Çinar and Ergen 2002, 2003, Çinar et al. 2003, Aguado and San Martín 2007, Abd-Elnaby and San Martín 2010, 2011). In Greece, polychaetes have been studied by various authors (e.g. Bellan 1964, Fassari 1982, Arvanitidis 1994, 2000, Simboura 1996, Simboura and Nicolaidou 2001, Antoniadou et al. 2004). However, the only studies in the Aegean Sea focussing specifically on Syllidae are those of Çinar (1999) and Çinar and Ergen (2002) from the Turkish Aegean coasts. Polychaetes of the Mediterranean coast of Israel have been studied by Monro (1937), Tebble (1959), Fauvel (1955, 1957), Ben-Eliahu (1976a, 1976b), Ben-Eliahu and Golani (1990) and Ben-Eliahu and Fiege (1995) and syllids in particular by Harlock and Laubier (1966) and Ben-Eliahu (1977a, 1977b).

This paper gives an account of the syllid species encountered in the three sampling locations and provides detailed information on the morphology, distribution and ecology of those species recorded for the first time in the respective area. Furthermore, during this study it became clear that the distribution range of the genus *Prosphaerosyllis* San Martín, 1984 in the Mediterranean is outdated or confused. In addition, since several new species have recently been described in this genus (Çinar et al. 2011, Olivier et al. 2011) and were also identified in the present material, an update on the distribution of the genus *Prosphaerosyllis* in the Mediterranean and an updated identification key are provided at the end of this paper.

## Material and methods

### Specimen collection and processing

Specimens from Israel were collected on 31 May 2009 and 11 Oct 2009 in Haifa Bay, (Israel, eastern Mediterranean Sea) from soft sediments of mixed grain sizes in shallow waters (Table 1). Sediment samples were taken with a Van-Veen grab (KAHLSICO, model WA265/SS214) 32×35cm, volume 20 l, penetration 20 cm. The sediment was preserved in buffered formalin 10% for 3–7 days, then sieved through a 250 µm mesh sieve and subsequently stored in 70% ethanol. In this study, only a subset of the collected material is presented.

Specimens from Crete were collected in September 2007 and June 2008 from two sites in northern Crete characterized by a continuous hard bottom habitat with dense algal coverage and a moderate wave exposure (Table 1). At each site, two vertical transects with sampling depths at 1 m, 5 m, 10 m, 15 m and 20 m were defined and five replicates were taken from each transect and depth. Samples were collected by means of SCUBA diving according to the NaGISA protocol (Iken and Konar 2003). A plexiglas frame (25 × 25 cm) with a net of 0.5 mm mesh size attached to its top opening was placed onto the rock and the surface within the frame was scraped off. The sample was collected by a manually operated suction device, supplied by air from an extra scuba tank. Large particles (>2 cm) were collected manually after suction. The samples were subsequently washed through a 0.5 mm mesh sieve, fixed and preserved in 99% ethanol.

Specimens were examined under an Olympus SZx12 stereomicroscope and an Olympus BX50 microscope and identified by employing the most recent literature on Syllidae (e.g. Nygren 2004, San Martín 2003, 2005, San Martín and Hutchings 2006). Illustrations in pencil were made by means of a drawing tube, subsequently scanned, imported into a graphic program (GIMP), re-drawn and saved as a vector graphic. All specimens are deposited in the invertebrate collection of the Institute of Marine Biology and Genetics, Hellenic Centre for Marine Research. Comparative material has been loaned by the Zoologisches Museum and Institut, Universität Hamburg, Germany, Ege University, Izmir, Turkey and the Muséum National d’Histoire Naturelle, Paris, France.

Information on habitat and global distribution of species was adopted from San Martín (2003), unless indicated otherwise, and updated with findings from this study. Information on species distribution among Mediterranean regions was adopted from Musco and Giangrande (2005) and updated according to recent literature and to findings from this study. Abbreviations for biogeographic regions used in the text are: MED (Mediterranean), WB (Western Basin), EB (Eastern Basin), CB (Central Basin), AD (Adriatic Sea), AS (Aegean Sea), BS (Black Sea), LB (Levantine Basin), following Arvanitidis et al. 2002 who modified Por’s (1989) system.

### Electronic publication

This manuscript was prepared in a Virtual Research Environment (Scratchpads) allowing for rapid and simultaneous publication of the results in print as well as electronically in a semantically enhanced form (Blagoderov et al. 2010, Penev et al. 2010). This publication and all supplementary data (tables, figures, taxon information) are also available under a Creative Commons license on the Polychaete Scratchpads (http://polychaetes.marbigen.org).

The underlying dataset of this study has been published under a Creative Commons license according to the Pensoft Data Publishing Policies and Guidelines for Biodiversity Data (Penev et al. 2011) and are available through the GBIF Integrated Publishing Toolkit hosted by Pensoft (http://ipt.pensoft.net/ipt/resource.do?r=easternmedsyllids). The data are furthermore available in Darwin Core Archive format, a simple and extensible schema for sharing biodiversity data which has been developed by the Global Biodiversity Information Facility (GBIF, http://www.gbif.org/informatics/standards-and-tools/publishing-data/data-standards/darwin-core-archives/) to allow easy and rapid mobilisation of species occurrence data through the internet. Darwin Core Archives are essentially a set of text files stored together with an XML descriptor file which describes the structure of the data files. Data are described through the Darwin Core schema, allowing for their usage within the semantic web. This new type of data publishing allows data to be indexed and discoverable through global biodiversity infrastructures such as GBIF or other data repositories, allows data to be integrated and compared with other datasets and ensures proper accreditation of the data provider (Penev et al. 2011). Additionally, the data have been deposited in the Dryad Data Repository (http://www.datadryad.org) and can be accessed at doi: 10.5061/dryad.4b7k408g.

## Results

**Figure 2.**
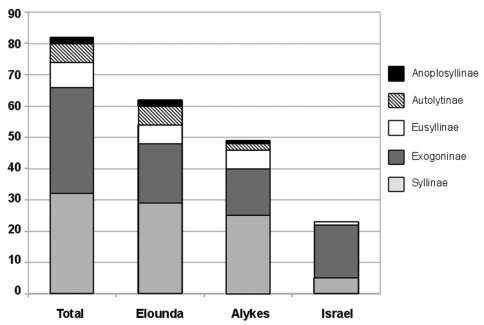
Numbers of species per subfamily at the three locations and in total.

**Figure 3.**
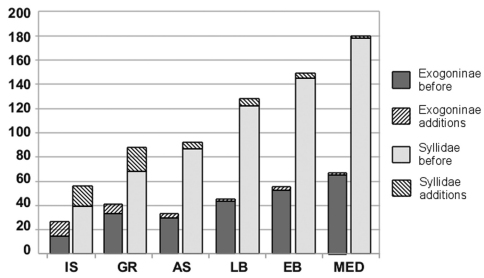
Numbers of additions of Syllidae and Exogoninae to various regions of the Mediterranean. IS=Israel, GR=Greece, AS=Aegean Sea, LB=Levantine Basin, EB=Eastern Basin, MED=Mediterranean.

Examination of a total of 111 samples yielded 82 syllid species (Table 2), of which 49 were found in Alykes (Crete), 62 in Elounda (Crete) and 23 in Haifa Bay (Israel). Species of all subfamilies have been found in the stations in Crete, with the majority (80%) of species belonging to Syllinae and Exogoninae, whereas the samples from Israel did not contain any specimens of Anoplosyllinae or Autolytinae, and 73% of the examined species belong to the small-sized Exogoninae (Fig. 2). The material yielded a number of species reported for the first time in the studied areas: Twenty species are reported for the first time in Greek waters, of these, six are new additions to the Aegean fauna. Seventeen species are newly reported for the Israeli coast, of these, 4 are also new records for the Levantine Basin. The studied material yielded also 4 species which are new additions to the eastern Mediterranean and 2 to the Mediterranean fauna (Table 2, Fig. 3). Information on morphology, distribution and ecology of the newly recorded species are given below.

**Table 2.** Species occurences at sampling stations. Years and replicates have been pooled. Transects sampled in Alykes and Elounda have been combined. †= new record for Greece, ‡= new record for the Aegean, §= new record for Israel, |= new record for the Levantine Basin, ¶= new record for the eastern Mediterranean, #= new record for the Mediterranean.

Species	CALA-1 CALB-1	CALA-5 CALB-5	CALA-10 CALB-10	CALA-15 CALB-15	CALA-20 CALB-20	CELA-1 CELB-1	CELA-5 CELB-5	CELA-10 CELB-10	CELA-15 CELB-15	CELA-20 CELB-20	ALA-IL-1	ALA-IL-2	ALA-IL-5	ALA-IL-7	ALA-IL-8	ALA-IL-9	ALA-IL-10
*Branchiosyllis exilis* (Gravier, 1900)		**+**					**+**	**+**						**+**			
*Brania arminii* (Langerhans, 1881)		**+**			**+**												
*Brania pusilla* (Dujardin, 1851)						**+**		**+**									
*Eurysyllis tuberculata* Ehlers, 1864	**+**			**+**		**+**	**+**	**+**	**+**	**+**				**+**			
*Eusyllis assimilis* Marenzeller, 1875			**+**			**+**	**+**	**+**	**+**								
*Eusyllis lamelligera* Marion & Bobretzky, 1875					**+**												
*Exogone dispar* (Webster, 1879)	**+**				**+**		**+**	**+**	**+**								
*Exogone naidina* Örsted, 1845			**+**	**+**	**+**	**+**	**+**	**+**	**+**	**+**							
*Exogone rostrata* Naville, 1933				**+**													
*Exogone verugera* (Claparède, 1868)		**+**		**+**	**+**		**+**	**+**	**+**								
*Haplosyllis spongicola* (Grube, 1855)	**+**	**+**	**+**	**+**		**+**	**+**	**+**	**+**								
*Myrianida convoluta* (Cognetti, 1953)			**+**			**+**		**+**	**+**								
*Myrianida edwarsi* (Saint-Joseph, 1887)						**+**											
*Myrianida inermis* (Saint-Joseph, 1887) †, ‡						**+**											
*Myrianida prolifera* (O.F. Müller, 1788)						**+**		**+**									
*Myrianida quindecimdentata* (Langerhans, 1884) †	**+**		**+**			**+**	**+**	**+**									
*Nudisyllis divaricata* (Keferstein, 1862)						**+**		**+**	**+**								
*Odontosyllis ctenostoma* Claparède, 1868			**+**			**+**	**+**	**+**	**+**								
*Odontosyllis fulgurans* (Audouin & Milne Edwards, 1834)		**+**	**+**	**+**	**+**		**+**	**+**	**+**	**+**							
*Odontosyllis gibba* Claparède, 1863		**+**	**+**				**+**	**+**	**+**	**+**							
*Opisthosyllis brunnea* Langerhans, 1879 †						**+**	**+**										
*Paraehlersia ferrugina* (Langerhans, 1881)	**+**	**+**	**+**	**+**	**+**		**+**	**+**	**+**	**+**							
*Parapionosyllis brevicirra* Day, 1954							**+**							**+**			
*Parapionosyllis elegans* (Pierantoni, 1903) §														**+**			**+**
*Parapionosyllis minuta* (Pierantoni, 1903)														**+**			**+**
*Parexogone hebes* Cognetti, 1955 §											**+**			**+**			
*Perkinsyllis augeneri* (Hartmann-Schröder, 1979) §, |, ¶, #														**+**			
*Plakosyllis brevipes* Hartmann-Schröder, 1956			**+**		**+**				**+**								
*Prosphaerosyllis adelae* San Martín, 1984 §, |, ¶												**+**		**+**	**+**		**+**
*Prosphaerosyllis campoyi* (San Martín, Acero, Contonente & Gómez, 1982)†								**+**									
*Prosphaerosyllis chauseyensis* Olivier et al. 2011 §, |, ¶, #											**+**	**+**	**+**	**+**	**+**	**+**	**+**
*Prosphaerosyllis longipapillata* (Hartmann-Schröder, 1979) §														**+**			
*Prosphaerosyllis marmarae* Çinar et al. 2011 §												**+**		**+**	**+**		
*Prosphaerosyllis xarifae* (Hartmann-Schröder, 1960) †,§							**+**	**+**									**+**
*Salvatoria alvaradoi* (San Martín, 1984) †, ‡		**+**	**+**			**+**	**+**	**+**	**+**	**+**							
*Salvatoria clavata* (Claparède, 1863)	**+**	**+**	**+**	**+**	**+**	**+**	**+**	**+**	**+**	**+**							
*Salvatoria euritmica* (Sardà, 1984) †	**+**				**+**	**+**		**+**	**+**	**+**							
*Salvatoria limbata* (Claparède, 1868)	**+**	**+**	**+**	**+**	**+**	**+**	**+**	**+**	**+**	**+**							
*Salvatoria neapolitana* (Goodrich, 1930) †						**+**			**+**	**+**							
*Salvatoria vieitezi* (San Martín, 1984) †	**+**	**+**	**+**	**+**	**+**	**+**	**+**	**+**	**+**	**+**							
*Salvatoria yraidae* (San Martín, 1984) †			**+**	**+**	**+**		**+**	**+**	**+**	**+**							
*Sphaerosyllis austriaca* Banse, 1959	**+**			**+**			**+**	**+**									
*Sphaerosyllis bulbosa* Southern, 1914 §														**+**			**+**
*Sphaerosyllis glandulata* Perkins, 1981 †, §									**+**					**+**	**+**		**+**
*Sphaerosyllis gravinae* Somaschini & San Martín, 1994 §, |, ¶															**+**		
*Sphaerosyllis hystrix* Claparède, 1863		**+**				**+**		**+**						**+**			
*Sphaerosyllis levantina* Faulwetter et al. 2011														**+**			
*Sphaerosyllis pirifera* Claparède, 1868	**+**	**+**	**+**	**+**	**+**	**+**	**+**	**+**	**+**	**+**							
*Sphaerosyllis* sp. [San Martín, 2003]														**+**			
*Sphaerosyllis taylori* Perkins, 1981 §											**+**	**+**		**+**	**+**		**+**
*Sphaerosyllis thomasi* San Martín, 1984 §														**+**			
*Syllides edentatus* Westheide, 1974 †							**+**	**+**									
*Syllides fulvus* (Marion & Bobretzy, 1875)					**+**	**+**	**+**	**+**	**+**								
*Syllides japonicus* Imajima, 1966 ‡							**+**		**+**								
*Syllis alternata* Moore, 1908	**+**		**+**	**+**	**+**	**+**	**+**	**+**	**+**	**+**							
*Syllis armillaris* (O.F. Müller, 1771)	**+**	**+**	**+**	**+**		**+**	**+**	**+**	**+**								
*Syllis beneliahuae* (Campoy & Alquézar, 1982)	**+**	**+**	**+**	**+**	**+**	**+**		**+**		**+**							
*Syllis columbretensis* (Campoy, 1982)	**+**	**+**	**+**	**+**	**+**	**+**	**+**	**+**	**+**	**+**							
*Syllis compacta* Gravier, 1900 †	**+**	**+**		**+**	**+**		**+**	**+**	**+**	**+**							
*Syllis corallicola* Verrill, 1900	**+**	**+**	**+**	**+**	**+**	**+**	**+**	**+**	**+**	**+**							
*Syllis cruzi* Núnez & San Martín, 1991 †, ‡					**+**			**+**									
*Syllis ferrani* Alós & San Martín, 1987						**+**	**+**	**+**		**+**							
*Syllis garciai* (Campoy, 1982)	**+**	**+**	**+**	**+**	**+**	**+**		**+**	**+**	**+**		**+**		**+**			
*Syllis gerlachi* (Hartmann-Schröder, 1960)	**+**	**+**	**+**	**+**	**+**	**+**	**+**	**+**	**+**	**+**							
*Syllis gerundensis* (Alós & Campoy, 1981) †, ‡		**+**			**+**	**+**	**+**										
*Syllis gracilis* Grube, 1840	**+**					**+**		**+**									
*Syllis hyalina* Grube, 1863	**+**	**+**		**+**	**+**	**+**	**+**	**+**	**+**	**+**							
*Syllis jorgei* San Martín & López, 2000 §	**+**				**+**	**+**	**+**							**+**			
*Syllis krohnii* Ehlers, 1864		**+**			**+**	**+**	**+**	**+**	**+**	**+**							
*Syllis parapari* San Martín & López, 2000					**+**												
*Syllis prolifera* Krohn, 1852	**+**	**+**	**+**	**+**	**+**	**+**	**+**	**+**	**+**	**+**							
*Syllis pulvinata* (Langerhans, 1881) †, ‡						**+**	**+**		**+**								
*Syllis rosea* (Langerhans, 1879)	**+**																
*Syllis tyrrhena* (Licher & Kuper, 1998) †, ‡, ¶								**+**									
*Syllis variegata* Grube, 1860						**+**	**+**		**+**								
*Syllis westheidei* San Martín, 1984 †				**+**													
*Synmerosyllis lamelligera* (Saint-Joseph, 1887)	**+**			**+**		**+**			**+**								
*Trypanosyllis aeolis* Langerhans, 1879									**+**								
*Trypanosyllis coeliaca* Claparède, 1868 §	**+**	**+**	**+**			**+**	**+**	**+**	**+**					**+**			
*Trypanosyllis zebra* (Grube, 1860)	**+**					**+**		**+**									
*Virchowia clavata* Langerhans, 1879						**+**											
*Xenosyllis scabra* (Ehlers, 1864)	**+**			**+**	**+**	**+**	**+**	**+**	**+**								

## New records

### Subfamily Anoplosyllinae Aguado and San Martín, 2009

#### Genus Syllides

Ørsted, 1845

##### Type species.

*Syllides longocirrata* Ørsted, 1845

#### Syllides edentatus

Westheide, 1974

http://species-id.net/wiki/Syllides_edentatus

Syllides japonica edentata Westheide, 1974a: 81, figs 36e, 37;Campoy 1982: 320; San Martín et al. 1985: 32.
Syllides edentatus : San Martín 1984b: 143, fig. 27; 2003: 143, fig. 70; Çinar 1999: 211, fig. 4.86; Çinar and Gambi 2005: 753.

##### Material examined.

Elounda, Crete, Greece: CELA-5b-08 (2 ind.), CELA-5d-08 (2 ind.) [coll. 12.6.2008]; CELB-10c-07 (1 ind.) [coll. 27.9.2007].

##### Type locality.

Galápagos Islands (Pacific Ocean).

##### Distribution.

Galápagos Islands, north-east Pacific, Atlantic, Mediterranean Sea: WB, AS. New record for the Greek coast.

##### Habitat.

Shallow subtidal depths, in sandy and muddy sediments, among photophilic algae and *Zostera* beds, in vermetid reefs.

#### Syllides japonicus

Imajima, 1966

http://species-id.net/wiki/Syllides_japonicus

Syllides japonicus Imajima, 1966: 112, figs 36a–h; Banse 1971: 1477, fig. 5; San Martín 2003: 142, fig. 69; San Martín and Hutchings 2006: 360, figs 86c–f, 87a–e.
Syllides cf. *japonicus*:San Martín 1984b: 139, fig. 26.

##### Material examined.

Elounda, Crete, Greece: CELA-15a-07 (1 ind.) [coll. 26.9.2007]; CELA-5d-08 (1 ind.), CELB-15d-08 (1 ind.) [coll. 12.6.2008].

##### Type locality.

Japan (Pacific Ocean).

##### Distribution.

Japan, Australia (San Martín and Hutchings 2006), Mediterranean Sea: WB, AS, LB (Abd-Elnaby and San Martín 2010). New record for the Aegean Sea.

##### Habitat.

Shallow subtidal depths, in sandy and muddy sediments, on rocks with algal cover, among *Posidonia oceanica* rhizomes.

### Subfamily Autolytinae Langerhans, 1879

#### Genus Myrianida

Milne Edwards, 1845

##### Type species.

*Myrianida fasciata* Milne Edwards, 1845

#### Myrianida inermis

(Saint-Joseph, 1887)

http://species-id.net/wiki/Myrianida_inermis

Autolytus inermis Saint-Joseph, 1887: 237, pl. 12, fig. 117; Gidholm 1967: 193, fig. 22; Campoy 1982: 235; San Martín 1994: 274, fig. 4; 2003: 487, figs 267a, c–e; Hartmann-Schröder 1996: 182.
Autolytus (Autolytides) inermis : Fauvel 1923: 322, figs 123h–k.
Myrianida inermis : Nygren 2004: 135, figs 65a–e.

##### Material examined.

Elounda, Crete, Greece: CELB-1e-07 (1 ind.) [coll. 29.9.2007].

##### Type locality.

Dinard, France (north-east Atlantic Ocean).

##### Distribution.

North-east Atlantic, north-west Atlantic (San Martín 1994), north-east Pacific (Nygren 2004), Arctic (Ramos et al. 2010). Mediterranean Sea: WB, AS. New record for the Aegean Sea.

##### Habitat.

Until 100m depth, on rocks among algae and hydrozooans, in coralligenous substrates (Nygren 2004, San Martín 2003).

#### Myrianida quindecimdentata

(Langerhans, 1884)

http://species-id.net/wiki/Myrianida_quindecimdentata

Autolytus quindecimdentatus Langerhans, 1884: 249, pl. 15, figs 3a–b; Gidholm 1967: 195, fig. 23; Ben-Eliahu 1977a: 86, fig. 13; Campoy 1982: 241; San Martín 1984b: 417, fig. 113; 2003: 494, figs 272a–d, 273a–b; Núñez and San Martín 1996: 213, figs 5k–m; Hartmann-Schröder 1996: 185; Çinar 1999: 63, fig. 4.8; Çinar et al. 2003: 747.
Autolytus lugens Saint-Joseph, 1887: 234, pl. 12, fig. 116; Fauvel, 1923: 318, fig. 122g; Cognetti 1961: 304.
Odontosyllis longicornis Hartmann-Schröder, 1960: 98, figs 101–104.
Myrianida quindecimdentata : Nygren 2004: 135, figs 77a–e.

##### Material examined.

Alykes, Crete, Greece: CALA-10c-08 (4 ind.) [coll. 17.6.2008]; CALA-1b-08 (1 ind.), CALB-1c-08 (1 ind.), CALB-1d-08 (1 ind.) [coll. 18.6.2008]. Elounda, Crete, Greece: CELB-5e-07 (1 ind.) [coll. 27.9.2007]; CELB-1a-07 (2 ind.), CELA-1d-07 (2 ind.), CELB-1e-07 (5 ind.) [coll. 29.9.2007]; CELA-10b-08 (1 ind.) [coll. 11.6.2008]; CELB-1a-08 (1 ind.), CELB-1b-08 (1 ind.), CELA-5d-08 (1 ind.) [coll. 12.6.2008].

##### Type locality.

Madeira (Atlantic Ocean).

##### Distribution.

East and west Atlantic (European and African coasts, Cuba), north-east Pacific, Red Sea (San Martin 1994, Nygren 2004). Mediterranean Sea: WB, CB, AD, AS, LB. New record for the Greek coast.

##### Habitat.

Subtidal depths, on biogenic calcareous substrates, among photophilic and sciaphilic algae and *Posidonia oceanica* rhizomes, endobiontic in sponges (Nygren 2004, San Martín 2003).

### Subfamily Eusyllinae Malaquin, 1893

#### Genus Perkinsyllis

San Martín, López & Aguado, 2009

##### Type species.

*Pionosyllis longisetosa* Hartmann-Schröder, 1965

#### Perkinsyllis augeneri

(Hartmann-Schröder, 1979)

http://species-id.net/wiki/Perkinsyllis_augeneri

Pionosyllis augeneri Hartmann-Schröder, 1979: 98, figs 119–125; 1980a: 52; 1981: 32, fig. 52 (Non Hartmann-Schröder 1991: 35); San Martín and Hutchings 2006: 326. figs 57a–j, 58a–f.
Perkinsyllis augeneri : San Martín et al. 2009: 26.

##### Material examined.

Haifa Bay, Israel: ALA-IL-7 (7 ind.) [coll. 11.10.2009].

##### Type locality.

Boone, west Australia.

##### Distribution.

Australia, New Zealand. Mediterranean Sea: LB. New record for the Mediterranean Sea.

##### Habitat.

Intertidal and shallow subtidal depths, in coarse coralline sand, in muddy sand and seagrass beds (San Martín and Hutchings 2006).

##### Taxonomic characters.

Prostomium pentagonal with 4 eyes in trapezoidal arrangement, posterior pair closer together than anterior one. Palps longer than prostomium, basally fused. Antennae cylindrical, smooth, longer than prostomium and palps. Tentacular cirri similar to antennae but slightly longer. Dorsal cirri of some anterior segments slender, longer than body width, some shorter, in midbody alternating short and long cirri, posteriorly all shorter than body width. Parapodia with 9–10 falcigers per fascicle anteriorly, 6–7 posteriorly. Shafts smooth or slightly serrated. Blades with marked dorso-ventral gradation (dorsal ones 3 times longer than ventral ones), coarsely serrated, with small subdistal tooth. After proventriculum, dorsal blades unidentate, elongated, spiniger-like, twice as long as anteriorly, ventral blades stout, with strong serration, especially basally. Dorsal simple chaeta first appearing on midbody, blunt, subdistally serrated. Ventral simple chaetae posteriorly, bidentate, equally sized teeth forming a right angle, some long spines subdistally. Paired aciculae anteriorly, single ones posteriorly, with rounded, slightly enlarged tip. Pharynx through 4 chaetigers, pharyngeal tooth located anteriorly. Proventricle through 5 chaetigers with ca. 20–22 muscle cell rows.

##### Remarks.

The subfamilial affiliation of *Perkinsyllis augeneri* has not yet been fully resolved. In recent molecular phylogenies the species groups either within Exogoninae or as a sister group, and forms a sister clade of Eusyllinae in all analyses (Aguado and Bleidorn 2010, Aguado et al. 2007).

The morphological characters of the Mediterranean individuals agree well with the description of San Martín and Hutchings (2006) from Australia. Therefore, a detailed description of the specimens is unnecessary here. The Mediterranean specimens show slight differences from the description of the Australian ones in the length of the pharynx (6–7 chaetigers in Australian specimens vs 5 in Mediterranean ones), and the number of falcigers per bundle in anterior chaetigers (ca. 15 in Australian specimens vs ca. 10 in Mediterranean ones). These differences might however be attributed to fixation and / or individual variation.

Until now, the species had been known only from north-west Australia and New Zealand, while the record from the Carribean Sea (Hartmann-Schröder 1980a) is assumed to be a different species (San Martín et al. 2009). The present findings thus extend the distribution range of the species to the eastern Mediterranean Sea. Since there are no intermediate records of the species from the Indian Ocean or Red Sea, this disjunct distribution suggests a potential human-induced introduction of the species to the Mediterranean Sea by vectors such as ballast water or fouling fauna on the hulls of ships. However, since the polychaete fauna of the Indian Ocean, Red Sea and eastern Mediterranean Sea is understudied, the species might have a truly circumtropical distribution. This is the second record of an Australian syllid species for the Mediterranean Sea (after *Prosphaerosyllis longipapillata* (Hartmann-Schröder, 1979), recorded for the first time in 2003 in Cyprus (Çinar et al. 2003)).

### Subfamily Exogoninae Langerhans, 1879

#### Genus Parapionosyllis

Fauvel, 1923

##### Type species.

*Pionosyllis gestans* Pierantoni, 1903

#### Parapionosyllis elegans

(Pierantoni, 1903)

http://species-id.net/wiki/Parapionosyllis_elegans

Pionosyllis elegans Pierantoni, 1903: 236, pl. X, fig. 2: pl. XI, fig. 27.
Parapionosyllis elegans : Fauvel 1923: 291, figs 111d–e; San Martín 1984b: 194, figs 42–43; 2003: 285, fig. 156; Çinar 1999: 127, fig. 4.40; Çinar et al. 2003: 755.

##### Material examined.

Haifa Bay, Israel: ALA-IL-7 (11 ind.), ALA-IL-10 (45 ind.) [coll. 11.10.2009].

##### Type locality.

Gulf of Naples (western Mediterranean Sea).

##### Distribution.

North-east Atlantic (Iberian Peninsula). Mediterranean Sea: WB, CB, AD, AS, LB. New record for the Israeli coast.

##### Habitat.

Until 30 m depth (San Martín 1984b), in medium to coarse sands.

#### Genus Prosphaerosyllis

San Martín, 1984

##### Type species.

*Sphaerosyllis xarifae* Hartmann-Schröder, 1960

#### Prosphaerosyllis adelae

San Martín, 1984

http://species-id.net/wiki/Prosphaerosyllis_adelae

Sphaerosyllis (Prosphaerosyllis) adelae San Martín, 1984a: 376, figs 1–4.
Prosphaerosyllis adelae : San Martín: 2003: 220, fig. 116.

##### Material examined.

Haifa Bay, Israel: ALA-IL-7 (11 ind.) [coll. 31.5.2009]; ALA-IL-7 (6 ind.), ALA-IL-10 (2 ind.) [coll. 11.10.2009].

##### Type locality.

Balearic Islands (western Mediterranean Sea).

##### Distribution.

Mediterranean Sea: WB, LB. New record for the eastern Mediterranean Sea.

##### Habitat.

Until 13 m depth, in coarse sands, among *Posidonia oceanica* rhizomes.

#### Prosphaerosyllis campoyi

(San Martín, Acero, Contonente & Gomez, 1982)

http://species-id.net/wiki/Prosphaerosyllis_campoyi

Sphaerosyllis campoyi San Martín Acero, Contonente and Gomez, 1982: 175, fig. 2; San Martín et al. 1985: 30, figs 3c–d; Çinar 1999: 146, fig. 4.50; Çinar et al. 2003: 756.
Sphaerosyllis (Prosphaerosyllis) campoyi : Núñez et al. 1992: 51.
Prosphaerosyllis campoyi : San Martín, 2003: 222, figs 117–118.

##### Material examined.

Elounda, Crete, Greece: CELA-10a-07 (1 ind.) [coll. 27.9.2009]; CELA-10b-08 (1 ind.) [coll. 11.6.2008].

##### Type locality.

Andalusia, Spain (western Mediterranean Sea).

##### Distribution.

North-east Atlantic (Iberian Peninsula, Canary Islands), Mediterranean Sea: WB, AS, LB. New record for the Greek coast.

##### Habitat.

Until 70 m depth (Çinar et al. 2003), on rocks among algae, on coralligenous substrates, in medium to coarse sands with organic material.

##### Remarks.

The specimens agree well with the description of San Martín (2003), except for having longer dorsal papillae (15 µm), especially posteriorly.

#### Prosphaerosyllis chauseyensis

Olivier, Grant, San Martín, Archambault & McKindsey, 2011

http://species-id.net/wiki/Prosphaerosyllis_chauseyensis

Figs 4 5

Prosphaerosyllis chauseyensis Olivier et al., 2011, figs 1–3a, b.

##### Material examined.

Haifa Bay, Israel: ALA-IL-8 (12 ind.) [coll. 31.5.2009]; ALA-IL-1 (23 ind.), ALA-IL-2 (4 ind.), ALA-IL-5 (1 ind.), ALA-IL-7 (73 ind.), ALA-IL-8 (24 ind.), ALA-IL-9 (68 ind.), ALA-IL-10 (99 ind.) [coll. 11.10.2009].

##### Comparative material examined.

*Sphaerosyllis brevicirra* Hartmann-Schröder, 1960 (Zoological Museum Hamburg, Holotype P-17566, Ghardaqa, Red Sea: 1 individual [Label: *Sphaerosyllis brevicirra* n. sp., Ghardaqa (Rot. Meer) (Typ), 29.3.56, coll. Remane/Schulz]); *Prosphaerosyllis chauseyensis* (Muséum National d’Histoire Naturelle, Paris, Holotype MNHN POLY TYPE 1524, Chausey Islands, France: 1 individual [Label: HOLOTYPE MNHN Paris 1524, Chausey, *Prosphaerosyllis* sp. A, (5 ind. for SEM +Holotype), C1AM et C3AV]).

**Figure 4.**
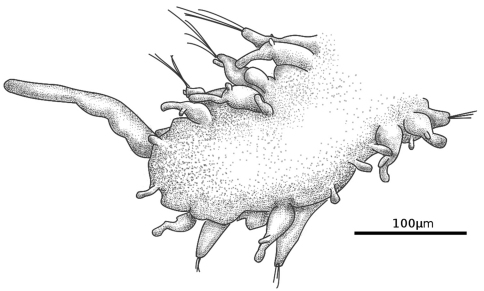
*Prosphaerosyllis chauseyensis*, pygidium (Israeli material).

**Figure 5.**
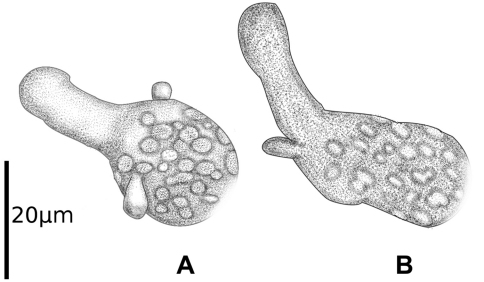
*Prosphaerosyllis chauseyensis*, anterior (A) and posterior (B) dorsal cirri (Israeli material).

##### Type locality.

Chausey Islands, Normandy (north-east Atlantic).

##### Distribution.

North-east Atlantic (Normandy), Mediterranean Sea: LB. New record for the Mediterranean Sea.

##### Habitat.

Until 13 m depth, in medium to very coarse sand.

##### Reproduction.

Three specimens collected at Station ALA-IL-8 on 31 May 2009 with egg capsules attached near dorsal cirri on midbody chaetigers.

##### Remarks.

The specimens from Israel agree well with the specimens from Normandy, however, the Mediterranean specimens differ from the Holotype in: a) Papillation pattern: each segment with one papilla between dorsal cirri and four papillae, situated dorso-laterally and ventro-laterally on each side of parapodium, most developed in posterior chaetigers, from mid-body additional papillae arranged in two very irregular lines along middle of dorsum, increasing in length towards posterior end (ca. 20 µm posteriorly). Ventrally 2 smaller (about half the size of dorsal papillae) papillae in middle of ventrum at posterior end of each segment. Specimens from Normandy have an irregular papillation pattern, but papillation is more distinct laterally, as in specimens from Israel; b) Length of anal cirri: about 125 µm, ca. 2.5–3 times length of posterior dorsal cirri (Fig. 4) (the anal cirri are broken in the holotype and the large lateral anal papillae might have been erroneously regarded as anal cirri in the original description). Specimens from both locations have anterior dorsal cirri with two papillae (dorsal and ventral) and posterior dorsal cirri only with dorsal papilla (Fig. 5, not reported by Olivier et al. 2011).

Individuals identified by Ben-Eliahu (1977a) as *Sphaerosyllis tetralix* Eliason, 1920, from the Gulf of Elat and the Mediterranean Sea might in fact belong to *Prosphaerosyllis chauseyensis*. The description and illustrations agree with many characteristics of *Prosphaerosyllis chauseyensis*, including the characteristic papilla on the dorsal cirri. However, Ben-Eliahu reports the species to have palps widely separated anteriorly (fused in *Prosphaerosyllis chauseyensis*), dorsum with four longitudinal rows of papillae (irregular rows in *Prosphaerosyllis chauseyensis*) and the proventriculum stretching through 4 chaetigers (5 in *Prosphaerosyllis chauseyensis*). The material of the species described by Ben-Eliahu could not be examined during this study, therefore it can only tentatively be assigned to *Prosphaerosyllis chauseyensis*.

#### Prosphaerosyllis longipapillata

(Hartmann-Schröder, 1979)

http://species-id.net/wiki/Prosphaerosyllis_longipapillata

Sphaerosyllis longipapillata Hartmann-Schröder, 1979: 106, figs 148–150; 1982: 71; 1984: 23; 1985: 71; 1986: 43; 1991: 40;Çinar et al. 2003: 757, fig. 5.
Prosphaerosyllis longipapillata : San Martín 2005: 61, figs 17a–g, 18a–h.

##### Material examined.

Haifa Bay, Israel: ALA-IL-7 (2 ind.) [coll. 11.10.2009].

**Comparative**

##### Material examined.

*Prosphaerosyllis longipapillata* (Hartmann-Schröder, 1979)(Department of Hydrobiology, Ege University, Izmir, Turkey, specimen reported in Çinar etal. 2003, Cyprus, Station D13: 1 individual [Label: *Prosphaerosyllis longipapillata*, Cyprus]).

##### Type locality.

Broome, north-west Australia.

##### Distribution.

Australia, Mediterranean Sea: LB. New record for the Israeli coast.

##### Habitat.

Intertidal to 466 m depth (San Martín 2005), euryoceous, found on hard substrates with *Sargassum vulgare* (Çinar etal. 2003, San Martín 2005).

##### Remarks.

The specimens from Israel agree well with the material and description of Çinar et al., 2003). However, both the material from Cyprus and Israel, as well as the description and illustrations of San Martín (2005), differ from Hartmann-Schröder’s (1979) original description by the presence of dorsal papillae on the anterior chaetigers. Hartmann-Schröder (1979) reports “four long, threadlike papillae at the height of the parapodia and from chaetiger 7 onwards in pairs in a dorsal row between the parapodia”. Furthermore, the Mediterranean material differs from the original description of *Prosphaerosyllis longipapillata* and from San Martín’s (2005) description by having alternating rows of long and short papillae on the dorsum (Çinar et al. 2003, fig. 5). These two characteristics are reported however for *Prosphaerosyllis bilineata* (Kudenov and Harris, 1995) from California. To determine the identity of the Mediterranean material and whether *Prosphaerosyllis bilineata* and *Prosphaerosyllis longipapillata* are different species or not, careful examination of all type material is needed.

#### Prosphaerosyllis marmarae

Çinar, Dagli & Açik, 2011

http://species-id.net/wiki/Prosphaerosyllis_marmarae

Prosphaerosyllis marmarae Çinar et al. 2011: 2118, figs 2–4.

##### Material examined.

Haifa Bay, Israel: ALA-IL-2 (3 ind.), ALA-IL-8 (12 ind.) [coll. 31.5.2009]; ALA-IL-7 (4 ind.) [coll. 11.10.2009].

##### Comparative material examined.

*Prosphaerosyllis marmarae* (Department of Hydrobiology, Ege University, Izmir, Turkey, Paratype: 1 individual [Label: *Prosphaerosyllis marmarae*, Paratype]). *Prosphaerosyllis laubieri* (Muséum National d’Histoire Naturelle, Paris, Holotype MNHN POLY TYPE 1525, Chausey Islands, France: 1 individual [Label: HOLOTYPE MNHN Paris 1525, Chausey B1 AM12, *Prosphaerosyllis* sp. B, Holotype et SEM]).

##### Type locality.

Erdek, Marmara Sea (eastern Mediterranean).

##### Distribution.

Mediterranean Sea: LB, Marmara Sea. New record for the Israeli coast.

##### Habitat.

Until 17 m depth, in muddy sand (Çinar et al. 2011), in coarse and mixed sand (this study).

##### Remarks

. The specimens from Israel agree with the material of Çinar et al. (2011), except for the absence of eyespots (might be de-colourised due to fixation). The recently described *Prosphaerosyllis laubieri* Olivier et al. 2011 is very similar to *Prosphaerosyllis marmarae*. Both species have eyespots, strongly papillated palps, short, retractile antennae and dorsal cirri, pharynx and proventriculum each through 4 segments and short (8–10 µm) blades of falcigers. These two species differ however in the following characteristics: a) *Prosphaerosyllis laubieri* has small, scattered papillae all over the dorsum, in *Prosphaerosyllis marmarae* they are restricted to the lateral margins, near the dorsal cirri; b) cirrostyles of antennae and dorsal cirri of *Prosphaerosyllis marmarae* are much shorter (1/4 of total length) than those of *Prosphaerosyllis laubieri* (1/3 of total length) and appear as small, retracted caps; c) dorsal cirri of *Prosphaerosyllis laubieri* possess a small papilla at distal end of cirrophore (not reported by Olivier et al. 2011); d) falcigerous blades of *Prosphaerosyllis marmarae* are stouter than those of *Prosphaerosyllis laubieri* and serrated only at their bases (serrated all along cutting edge in *Prosphaerosyllis laubieri*). *Prosphaerosyllis riseri* Perkins, 1981 from Florida shares with *Prosphaerosyllis marmarae* the shape of the dorsal cirri and antennae (short and strongly retracted), however, its palps are less densely papillated. *Prosphaerosyllis* sp. A (San Martín 1991b) from Cuba has strongly papillated palps, but no cirri on chaetiger 2 and longer dorsal cirri.

Specimens from the Red Sea described by Ben-Eliahu (1977a) as *Sphaerosyllis brevicirra* Hartmann-Schröder, 1960 do not belong to this species (see Discussion section), but might in fact belong to *Prosphaerosyllis marmarae*. The morphological characteristics of her specimens agree very well wth those of *Prosphaerosyllis marmarae* (papillated palps, presence of eyespots, minute (19.5 µm), retractile cirri, falcigerous blades short (7.8 µm), proventriculum longer than proboscis (through 4 segments), no discernible dorsal papillation). Differences can be found in the cutting edge of the falcigerous blades which are smooth in the Red Sea specimens, whereas those of *Prosphaerosyllis marmarae* are serrated. However, due to the size of the blades (8 µm) this is a feature difficult to observe under an optical microscope and might have been overlooked. The material of the species described by Ben-Eliahu was not examined during this study, therefore it can only tentatively proposed to be assigned to *Prosphaerosyllis marmarae*.

#### Prosphaerosyllis xarifae

(Hartmann-Schröder, 1960)

http://species-id.net/wiki/Prosphaerosyllis_xarifae

Sphaerosyllis xarifae Hartmann-Schröder, 1960: 103, figs 121–124; 1979: 103, figs 139–140; 1980b: 56; 1981: 37; 1984: 25; San Martín 1984b: 236, fig. 54; Çinar 1999: 166, fig. 4.62; Çinar et al. 2003: 760, fig. 6.
Sphaerosyllis sp.: San Martín and Alvarado 1981: 224, fig. 3.
Sphaerosyllis cf. xarifae :Campoy 1982: 279.
Sphaerosyllis (Prosphaerosyllis) xarifae :Núñez et al. 1992: 51.
Prosphaerosyllis xarifae : San Martín 2003: 225, figs 119–120; 2005: 60, figs 15a–f, 16a–f; Böggemann and Westheide 2004: 435; Fukuda et al. 2009: 1448, fig. 3.

##### Material examined.

Haifa Bay, Israel: ALA-IL-10 (5 ind.) [coll. 11.10.2009]. Elounda, Crete, Greece: CELA-10b-08 (1 ind.) [coll. 11.6.2008]; CELA-5c-08 (1 ind.) [coll. 12.6.2008].

##### Type locality.

Sarso, Red Sea.

##### Distribution.

Circumtropical, Mediterranean Sea: WB, CB, AS, LB. New record for both the Israeli and Greek coasts.

##### Habitat.

Until 40 m depth, euryoceous, among photophilic algae, in sand, mud, seagrasses, calcareous substrates (San Martín 2005).

##### Remarks.

Specimens from Israel agree well with the description of San Martín (2003) and Hartmann-Schröder (1960) except for having more elongated dorsal papillae, especially posteriorly (20 µm, Cretan specimens: 8 µm).

#### Genus Salvatoria

McIntosh, 1885

##### Type species.

*Salvatoria kerguelensis* McIntosh, 1885

#### Salvatoria alvaradoi

(San Martín, 1984)

http://species-id.net/wiki/Salvatoria_alvaradoi

Pseudobrania alvaradoi San Martín 1984b: 152, figs 28–29.
Salvatoria alvaradoi : San Martín 2003: 173, figs 87–88.

##### Material examined.

Alykes, Crete, Greece: CALB-10b-08 (5 ind.), CALB-10d-08 (2 ind.) [coll. 17.6.2008]; CALB-5a-08 (2 ind.) [coll 18.6.2008]. Elounda, Crete, Greece: CELA-15a-07 (3 ind.), CELB-20e-07 (1 ind.) [coll. 26.9.2007], CELA-10a-07 (1 ind.) [coll. 27.9.2007]; CELB-1a-07 (1 ind.) [coll. 29.9.2007]; CELB-10a-08 (1 ind.), CELA-10b-08 (4 ind.), CELB-10b-08 (3 ind.), CELB-10c-08 (1 ind.), CELA-20a-08 (1 ind.), CELA-20d-08 (3 ind.) [coll. 11.6.2008]; CELA-5a-08 (8 ind.), CELA-5c-08 (18 ind.), CELA-5d-08 (1 ind.), CELB-15a-08 (1 ind.), CELB-15c-08 (9 ind.) [coll. 12.6.2008].

##### Type locality.

Balearic Islands (western Mediterranean Sea).

##### Distribution.

Mediterranean Sea: WB, CB, AS, Sea of Marmara (Karhan et al. 2008). New record for the Aegean Sea.

##### Habitat.

Until 20 m depth, among algae with much sediment, among *Posidonia oceanica* rhizomes, in sediments with much organic material.

#### Salvatoria euritmica

Sardá, 1984

http://species-id.net/wiki/Salvatoria_euritmica

Pseudobrania euritmica Sardá, 1984: 10, fig. 1.
Grubeosyllis euritmica : San Martín 1991a: 718, figs 2c–d; Çinar 1999: 115, fig. 4.34; Çinar et al. 2003: 754.
Salvatoria euritmica : San Martín 2003: 169, figs 84–86; 2005: 53, figs 8a–g.
Pionosyllis yambaensis Hartmann-Schröder, 1990: 52, figs 18–22.

##### Material examined.

Alykes, Crete, Greece: CALB-20b-08 (1 ind.) [coll. 17.6.2008]; CALB-1d-08 (4 ind.) [coll. 18.6.2008]. Elounda, Crete, Greece: CELA-15c-07 (2 ind.) [coll. 27.9.2007]; CELB-1b-07 (4 ind.), CELA-1d-07 (1 ind.) [coll. 29.9.2007]; CELA-10b-08 (1 ind.), CELA-20c-08 (1 ind.) [coll. 11.6.2008]; CELB-15d-08 (1 ind.) [coll. 12.6.2008].

##### Type locality.

Strait of Gibraltar (western Mediterranean Sea).

##### Distribution.

Caribbean Sea, Australia, north-east Atlantic (Iberian Peninsula, Canary Islands), Mediterranean Sea: WB, AS, LB. New record for the Greek coast.

##### Habitat.

Until 20 m depth, on hard substrates between algae, in seagrass beds, on coralligenous substrates.

##### Remarks.

*Pionosyllis yambaensis* was synonymized with *Salvatoria euritmica* by San Martín (2005) based on examination of type material.

#### Salvatoria neapolitana

(Goodrich, 1930)

http://species-id.net/wiki/Salvatoria_neapolitana

Pionosyllis neapolitana Goodrich, 1930: 651, figs 1–12.
Pseudobrania neapolitana San Martín 1984b: 160, figs 31–32.
Grubeosyllis neapolitana : Jiménez et al. 1994: 52 figs 1–2; Böggemann and Westheide 2004: 430.
Salvatoria neapolitana : San Martín 2003: 182, fig. 94.
Pionosyllis subterranea Hartmann-Schröder, 1956: 89 figs 6–9.
Brania subterranea : Westheide 1974a: 10, fig. 6; 1974b: 87, figs 10, 42d–f.
Grubeosyllis subterranea : Núñez et al. 1992: 45.

##### Material examined.

Elounda, Crete, Greece: CELA-15a-07 (2 ind.), CELB-20c-07 (2 ind.) [coll. 26.9.2007]; CELB-15a-08: (5 ind.), CELB-15c-08 (1 ind.) [coll. 11.6.2008]; CELB-1d-08 (1 ind.) [coll. 12.6.2008].

##### Type locality.

Bay of Naples, Italy (western Mediterranean Sea).

##### Distribution.

Circumtropical, Mediterranean Sea: WB, AS (Çinar et al. 2008). New record for the Greek coast.

##### Habitat.

Until 20 m depth, in coarse sand, among photophilic algae.

##### Remarks.

*Pionosyllis subterranea* was synonymized with *Pionosyllis neapolitana* and transferred to *Grubeosyllis* by Jiménez et al. (1994). San Martín (2003) subsequently replaced the name *Grubeosyllis* with *Salvatoria*, which has priority over the former.

#### Salvatoria vieitezi

(San Martín, 1984)

http://species-id.net/wiki/Salvatoria_vieitezi

Pseudobrania vieitezi San Martín, 1984b: 160, figs 31–32.
Grubeosyllis vieitezi : San Martin 1991a: 718, fig. 2e–f; Çinar 1999: 117, fig. 4.35; Çinar et al. 2003: 754; López and San Martín 1997: 105, fig 3.
Salvatoria vieitezi : San Martín 2003: 184, figs 95–96.

##### Material examined.

Alykes, Crete, Greece: CALA-10d-08 (1 ind.), CALA-15c-08 (1 ind.), CALA-20c-08 (3 ind.,), CALB-20c-08 (1 ind.), CALB-20b-08 (1 ind.) [coll. 17.6.2008]; CALA-1b-08 (2 ind.), CALB-1b-08 (1 ind.), CALB-5a-08 (1 ind.) [coll. 18.6.2008]; CALB-20e-07 (1 ind.) [coll. 18.9.2007]; CALA-5c-07 (1 ind.) [coll. 19.9.2007]. Elounda, Crete, Greece: CELA-20d-07 (3 ind.) [coll. 26.9.2007]; CELA-10b-07 (1 ind.) [coll. 27.9.2007]; CELA-20c-08 (1 ind.), CELA-20d-08 (7 ind.) [coll. 11.6.2008]; CELA-5d-08 (1 ind.), CELB-15a-08 (1 ind.), CELB-1b-08 (5 ind.) [coll. 12.6.2008].

##### Type locality.

Balearic Islands (western Mediterranean Sea).

##### Distribution.

North-east Atlantic (Iberian Peninsula, Canary Islands), Caribbean, Mediterranean Sea: WB, CB, AS. New record for the Greek coast.

##### Habitat.

Until 30m depth, on rocky substrates among photophilic algae, as endobiont of sponges, among *Posidonia oceanica* rhizomes.

#### Salvatoria yraidae

(San Martín, 1984)

http://species-id.net/wiki/Salvatoria_yraidae

Pseudobrania yraidae San Martín, 1984b: 156, fig. 30.
Grubeosyllis yraidae : Çinar 1999: 121, fig. 4.37.
Salvatoria yraidae : San Martín 2003: 163, figs 80–81.

##### Material examined.

Alykes, Crete, Greece: CALB-10b-08 (1 ind.), CALB-15a-08 (1 ind.), CALB-20b-08 (3 ind.), CALB-20d-08 (1 ind.) [coll. 17.6.2008]. Elounda, Crete, Greece: CELA-15b-07 (1 ind.), CELA-15e-07 (2 ind.) [coll. 26.9.2007]; CELA-5c-07 (4 ind.) [coll. 27.9.2007]; CELA-10b-08 (3 ind.), CELB-10b-08 (8 ind.), CELB-10c-08 (1 ind.), CELA-20a-08 (1 ind.), CELA-20b-08 (1 ind.) [coll. 11.6.2008]; CELB-15a-08 (6 ind.), CELB-15c-08 (4 ind.), CELA-15d-08 (5 ind.), CELB-15d-08 (5 ind.) [coll. 12.6.2008].

##### Type locality.

Balearic Islands (western Mediterranean Sea).

##### Distribution.

Mediterranean Sea: WB, CB, AD, AS. New record for the Greek coast.

##### Habitat.

Until 20 m depth, in sandy substrates, on rocks among algae.

#### Genus Sphaerosyllis

Claparède, 1863

##### Type species.

*Sphaerosyllis hystrix* Claparède, 1863

#### Sphaerosyllis bulbosa

Southern, 1914

http://species-id.net/wiki/Sphaerosyllis_bulbosa

Sphaerosyllis bulbosa Southern, 1914: 20, plates I–II, figs 2a–g; Fauvel, 1923: 304, figs. 116h–r; Cognetti 1961: 30; Rullier 1972: 69; Campoy 1982: 276; Parapar et al. 1994: 98, fig. 4; Çinar et al. 2003: 756; San Martin 2003: 191, figs 98–99.
Sphaerosyllis (Sphaerosyllis) bulbosa : Hartmann-Schröder 1996: 175.

##### Material examined.

Haifa Bay, Israel: ALA-IL-7 (4 ind.), ALA-IL-10 (51 ind.) [coll. 11.10.2009].

##### Type locality.

Ireland (Atlantic Ocean).

##### Distribution.

North-east Atlantic, Arctic Sea (Ramos et al. 2010), New Caledonia (Rullier 1972). Mediterranean Sea: WB, CB, AD, AS, LB, BS (Surugiu 2005).New record for the Israeli coast.

##### Habitat.

Until 70 m depth, in sandy or muddy sediments, on calcareous substrates.

##### Remarks.

The examined material differs from the description of San Martín (2003) in having papillated palps.

#### Sphaerosyllis glandulata

Perkins, 1981

http://species-id.net/wiki/Sphaerosyllis_glandulata

Sphaerosyllis glandulata Perkins, 1981: 1123, figs 18–19; Uebelacker 1984: 33, figs 25–26; San Martín 1991a: 232; 2003: 193, fig. 100; Men et al. 1993: 31, fig. 8; Somaschini and San Martín 1994: 361, fig. 3; Çinar 1999: 152, fig. 4.53; San Martín and Bone 2001: 613.
Sphaerosyllis cf. *glandulata*: Ding and Westheide 2008: 131, figs. 5a–h.

##### Material examined.

Haifa Bay, Israel: ALA-IL-7 (1 ind.) [coll. 31.5.2009]; ALA-IL-7 (47 ind.), ALA-IL-10 (19 ind.) [coll. 11.10.2009]. Elounda, Crete, Greece: CELA-15d-08 (1 ind.) [coll. 12.6.2008].

##### Type locality.

Florida, Hutchinson Island.

##### Distribution.

West Atlantic (Florida, Caribbean Sea), China (Ding and Westheide 2008) Mediterranean Sea: WB, AD, AS, LB (Abd-Elnaby and San Martín 2010). New record for both the Israeli and Greek coasts.

##### Habitat.

Until 120 m depth, in calcareous habitats and fine to coarse sands, among photophilic algae.

##### Remarks.

The specimens from Israel differ from San Martín’s (2003) description in having papillated palps and a longer proventriculum (3–4 chaetigers vs 2 chaetigers in the Iberian material). Other characteristics, especially chaetal ones, agree well with former descriptions of *Sphaerosyllis glandulata*.

#### Sphaerosyllis gravinae

Somaschini & San Martín, 1994

http://species-id.net/wiki/Sphaerosyllis_gravinae

Sphaerosyllis gravinae Somaschini and San Martín, 1994: 358, figs 1–2; San Martín 2003: 188, fig. 97.

##### Material examined.

Haifa Bay, Israel: ALA-IL-8 (4 ind.) [coll. 31.5.2009].

##### Type locality.

Zannone Island, Italy (western Mediterranean Sea).

##### Distribution.

Mediterranean Sea: WB, AD, LB. New record for the eastern Mediterranean Sea.

##### Habitat.

Shallow subtidal depths, in medium to coarse sands, among algae.

#### Sphaerosyllis taylori

Perkins, 1981

http://species-id.net/wiki/Sphaerosyllis_taylori

Sphaerosyllis taylori Perkins, 1981: 1140, fig. 26; Uebelacker 1984: 29, figs 21–22; San Martín 1984b: 247, fig. 58; 2003: 206, fig. 108; Russell 1991: 71; Núñez et al. 1992: 49; Parapar et al. 1994: 99; Simboura 1996: 53, fig. 6; San Martín and Bone 2001: 614; Çinar 1999: 161, fig. 4.58; Ruíz-Ramírez and Salazar-Vallejo 2001: 131, fig. 6 (115–122); Çinar et al. 2003: 759; Liñero-Arana and Díaz-Díaz 2011: 9, figs 2.1–2.5 in online material.

##### Material examined.

Haifa Bay, Israel: ALA-IL-1 (1 ind.); ALA-IL-2 (33 ind.) [coll. 31.5.2009]; ALA-IL-7 (103 ind.), ALA-IL-10 (14 ind.) [coll. 11.10.2009].

##### Type locality.

Florida, Hutchinson Island.

##### Distribution.

North-east and north-west Atlantic (North Sea to Canary Islands, east coast of the U.S. to Venezuela), Pacific Ocean (Galápagos Islands) (Liñero-Arana and Díaz-Díaz 2011), Arctic Sea (Ramos et al. 2010), Mediterranean Sea: WB, CB, AD, AS, BS, LB (Abd-Elnaby and San Martín 2010). New record for the Israeli coast.

##### Habitat.

Shallow subtidal depths, in muddy to coarse sands with organic material, on rocks among photophilic or calcareous algae, among *Posidonia oceanica* rhizomes.

#### Sphaerosyllis thomasi

San Martín 1984

http://species-id.net/wiki/Sphaerosyllis_thomasi

Sphaerosyllis thomasi San Martín, 1984b: 250, fig. 59; 2003: 199, figs 103–104; Arvanitidis 1994: 80; Çinar 1999: 163, fig. 4.60.

##### Material examined.

Haifa Bay, Israel: ALA-IL-7 (2 ind.) [coll. 11.10.2009].

##### Type locality.

Balearic Islands (western Mediterranean Sea).

##### Distribution.

Mediterranean Sea: WB, CB, AD, AS, LB. New record for the Israeli coast.

##### Habitat.

Shallow subtidal depths, in muddy to coarse sands, among *Posidonia oceanica* rhizomes.

##### Remarks.

The examined specimens agree well with the description of San Martín (2003), especially in the chaeteal structures, but in the Israeli specimens the dorsal cirri are as long as parapodial lobes in posterior and midbody chaetigers and only slightly shorter than parapodial lobe in anterior chaetigers (dorsal cirri shorter than parapodial lobe in San Martín’s (2003) description).

### Subfamily Syllinae Grube, 1850

#### Genus Opisthosyllis

Langerhans, 1879

##### Type species.

*Opisthosyllis brunnea* Langerhans, 1879

#### Opisthosyllis brunnea

Langerhans, 1879

http://species-id.net/wiki/Opisthosyllis_brunnea

Opisthosyllis brunnea Langerhans, 1879: 541, fig. 7; Augener 1918: 274, text-fig. 25; Tebble 1956: 90, figs 5d–e; Day 1967: 253, figs 12.5 c–e. Hartmann-Schröder 1979: 86; 1980b: 48; 1981: 24; 1982: 58; 1991: 25, fig. 19; Fauchald 1977:20, fig. 5; San Martín 1984b: 311, figs 75–76; 2003: 330, fig. 183; Çinar 1999: 237, fig. 4.99; Amaral et al. 2005: 164, figs a–e on same page; Abd-Elnaby 2009: 15, plate 3-16, figs 3g–h.

##### Material examined.

Elounda, Crete, Greece: CELA-1d-07 (1 ind.) [coll. 29.9.2007], CELA-5d-08 (1 ind.) [coll. 12.6.2008].

##### Type locality.

Madeira (Atlantic Ocean).

##### Distribution.

Circumtropical. Mediterranean Sea: WB, CB, AS, LB. New record for the Greek coast.

##### Habitat.

Intertidal to shallow subtidal, on hard substrates (vermetid reefs, among photophilic algae), endobiont of sponges.

#### Genus Syllis

Lamarck, 1818

##### Type species.

*Syllis monilaris* Lamarck, 1818

#### Syllis alternata

Moore, 1908

http://species-id.net/wiki/Syllis_alternata

Syllis alternata Moore, 1908: 323; 1909: 321; Çinar 1999: 246, fig. 4.102; Çinar and Gambi 2005: 754; Çinar and Ergen 2003: 777.
Typosyllis alternata : Kudenov and Harris 1995: 83, fig. 1.32; Licher 2000: 253, figs 17p, 106; Imajima 2003: 163.

##### Material examined.

Alykes, Crete, Greece: CALB-15c-07 (1 ind.) [coll. 18.9.2007]; CALB-1a-07 (1 ind.) [coll. 19.9.2007]; CALA-10d-08 (2 ind.), CALA-15d-08 (1 ind.), CALB-20b-08 (1 ind.), CALA-20b-08 (2 ind.), CALA-20c-08 (5 ind.), CALB-20d-08 (6 ind.) [coll. 17.6.2008]. Elounda, Crete, Greece: CELB-20c-07 (1 ind.) [coll. 26.9.2007]; CELB-1a-07 (4 ind.) [coll. 29.9.2007]; CELA-10b-08 (1 ind.), CELB-10b-08 (1 ind.), CELA-10c-08 (1 ind.), CELB-10c-08 (1 ind.), CELA-10d-08 (2 ind.), [coll. 11.6.2008]; CELB-1a-08 (1 ind.), CELA-5b-08 (1 ind.), CELA-5d-08 (2 ind.), CELB-15c-08 (5 ind.) [coll. 12.6.2008].

##### Type locality.

Alaska (Pacific Ocean).

##### Distribution.

East Pacific (Alaska to Panama), west Atlantic (North Carolina to Cuba) (Capa et al. 2001), Japan (Imajima 2003), Indonesia (Aguado et al. 2008), Mediterranean: WB, CB, AS, LB. New record for the Greek coast.

##### Habitat.

Until 2500 m depth (Moore 1909), among *Posidonia oceanica* rhizomes, calcareous algae, corals and photophilic algae (San Martín 2003), in sandy and muddy sediments (Moore 1909).

#### Syllis compacta

Gravier, 1900

http://species-id.net/wiki/Syllis_compacta

Syllis (Typosyllis) compacta Gravier, 1900: 165, pl. 9, fig. 11, text-figs 35–38.
Syllis compacta : López et al. 1996: 110, fig 3; Çinar 1999: 263, fig. 4.113; San Martín 2003: 433, figs 238–239.
Syllis golfonovensis : San Martín 1984b: 395, fig. 104 (Non *Syllis golfonovensis* Hartmann-Schröder, 1962.

##### Material examined.

Alykes, Crete, Greece: CALB-1e-07 (1 ind.), CALA-5e-07 (1 ind.) [coll. 19.9.2007]; CALA-15c-08 (1 ind.), CALA-20b-08 (1 ind.), CALB-20b-08 (1 ind.), CALA-20c-08 (1 ind.) [coll. 17.6.2008]. Elounda, Crete, Greece: CELA-15b-07 (1 ind.), CELA-15e-07 (3 ind.), CELB-20a-07 (2 ind.) [coll. 26.9.2007]; CELA-10a-07 (1 ind.), CELA-10d-07 (1 ind.) [coll. 27.9.2007]; CELA-5c-07 (1 ind.), CELB-5d-07 (1 ind.) [coll. 29.9.2007]; CELA-5b-08 (1 ind.), CELA-5d-08 (2 ind.), CELB-15d-08 (3 ind.) [coll. 12.6.2008].

##### Type locality.

Red Sea.

##### Distribution.

Red Sea. Mediterranean Sea: WB, CB, AD, AS. New record for the Greek coast.

##### Habitat.

Shallow subtidal depths, on biogenic calcareous substrates, among photophilic algae and *Posidonia oceanica* rhizomes.

##### Remarks.

The species is regarded by many authors (e.g. Augener 1913, Fauvel 1919, Licher 2000) as a synonym of *Syllis variegata* Grube, 1860. Recent works (e.g. San Martín 2003, Çinar 2005) however, regard the two species as distinct, which is also supported by molecular analyses (Aguado et al. 2007).

#### Syllis cruzi

Núñez & San Martín, 1991

http://species-id.net/wiki/Syllis_cruzi

Syllis cruzi Núñez and San Martín, 1991: 238, figs 2a–j; Çinar and Ergen 2003: 780, fig. 2.
Typosyllis cruzi : Licher 2000: 169, fig. 75.

##### Material examined.

Alykes, Crete, Greece: CALB-20d-08 (1 ind.) [coll. 17.6.2008]. Elounda, Crete, Greece: CELB-10a-08 (1 ind.) [coll. 11.6.2008].

##### Type locality.

Canary Islands (Atlantic Ocean).

##### Distribution.

North-east Atlantic (Canary Islands), Mediterranean Sea: WB, CB, AD, AS, LB. New record for the Aegean Sea.

##### Habitat.

Until 115 m depth, on coralligenous substrates, among photophilic algae, endobiont of sponges.

#### Syllis gerundensis

(Alós & Campoy, 1981)

http://species-id.net/wiki/Syllis_gerundensis

Typosyllis gerundensis Alós and Campoy, 1981: 21, figs 1–3; Campoy 1982: 446, figs 55–56; Licher 2000: 171, fig. 77.
Syllis gerundensis : Çinar and Ergen 2003: 783; San Martín 2003: 419, figs 230–231.

##### Material examined.

Alykes, Crete, Greece: CALA-20b-08 (1 ind.), CALB-20b-08 (1 ind.) [coll. 17.6.2008]; CALA-5d-08 (2 ind.) [[coll. 18.6.2008]. Elounda, Crete, Greece: CELB-1e-07 (1 ind.) [coll. 29.9.2007]; CELB-1d-08 (1 ind.), CELA-5d-08 (3 ind.) [coll. 12.6.2008].

##### Type locality.

Columbretes Islands, Spain (western Mediterranean Sea).

##### Distribution.

Mediterranean Sea: WB, CB, AD, AS, LB.New record for the Aegean Sea.

##### Habitat.

Shallow subtidal depths, on calcareous grounds, sandy bottoms, among *Posidonia oceanica* rhizomes and photophilic algae, endobiont of sponges.

#### Syllis jorgei

San Martín & López, 2000

http://species-id.net/wiki/Syllis_jorgei

Syllis jorgei San Martín and López, 2000: 430, figs 4–6; San Martín 2003: 382, figs 208–210; Çinar and Ergen 2003: 785.
Typosyllis lutea : Campoy 1982: 428.
Syllis lutea : San Martín 1984b: 370, figs 94–95; Arvanitidis 1994: 101 (Non *Syllis lutea* Hartmann-Schröder, 1960).

##### Material examined.

Haifa Bay, Israel: ALA-IL-7 (3 ind.) [coll. 11.10.2009]. Alykes, Crete, Greece: CALA-20c-07 (1 ind.) [coll. 18.9.2007], CALB-1a-08 (1 ind.) [coll. 18.6.2008]. Elounda, Crete, Greece: CELB-1a-08 (1 ind.), CELA-1c-08 (1 ind.), CELB-5d-08 (1 ind.) [coll. 12.6.2008].

##### Type locality.

Columbretes Islands, Spain (western Mediterranean Sea).

##### Distribution.

East Atlantic (Canary Islands), Mediterranean Sea: WB, CB, AD, AS, LB. New record for the Israeli coast.

##### Habitat.

Until 145 m depth (Çinar and Ergen 2003), on biogenic calcareous structures, among *Posidonia oceanica* rhizomes and photophilic algae.

#### Syllis pulvinata

(Langerhans, 1881)

http://species-id.net/wiki/Syllis_pulvinata

Typosyllis pulvinata Langerhans, 1881: 97, 104; Licher 2000: 158, fig. 70.
Syllis pulvinata : Çinar and Ergen 2003: 787; San Martín 2003: 372, figs 202–204.
Syllis (Typosyllis) truncata mediterranea Ben-Eliahu, 1977a: 10, fig. 2.
Syllis mediterranea : San Martín, 1984b; 209, fig. 8.

##### Material examined.

Elounda, Crete, Greece: CELA-1b-08 (1 ind.), CELA-5c-08 (2 ind.), CELB-15d-08 (1 ind.) [coll. 12.6.2008].

##### Type locality.

Canary Islands (Atlantic Ocean).

##### Distribution.

North-east Atlantic (Cantabrian Sea to Canary Islands), Red Sea, Mediterranean: WB, CB, AD, AS, LB. New record for the Aegean Sea.

##### Habitat.

Shallow subtidal depths, on calcareous substrates (vermetid reefs), among photophilic algae, endobiont of sponges.

#### Syllis tyrrhena

(Licher & Kuper, 1998)

http://species-id.net/wiki/Syllis_tyrrhena

Typosyllis tyrrhena Licher and Kuper, 1998: 228, figs 1–4; Licher 2000: 140, figs 2, 14–16, 62–63; Kuper 2001: 58, figs 1a–b, 20–24. Amaral et al. 2005: 162, figs a–f on same page.
Syllis tyrrhena : San Martín 2003: 379, fig. 207.

##### Material examined.

Elounda, Crete, Greece: CELB-10b-08 (1 ind.) [coll. 11.6.2008].

##### Type locality.

Island of Elba, Italy (western Mediterranean Sea).

##### Distribution.

Brazil (Amaral et al. 2005), Mediterranean Sea: WB, AS. New record for the eastern Mediterranean Sea.

##### Habitat.

Until 13 m depth, in sandy substrates of mixed grain sizes (Licher and Kuper 1998), on rocks among algae (this study).

#### Syllis westheidei

San Martín, 1984

http://species-id.net/wiki/Syllis_westheidei

Syllis westheidei San Martín, 1984b: 403, figs 108–109; 2003: 436, figs 240–241; Çinar 1999: 310, fig. 4.141.
Typosyllis westheidei : Licher 2000: 111, fig. 51; Böggemann and Westheide 2004: 418.
Typosyllis variegata : Westheide 1974a: 51, figs 21–22. (Non *Syllis variegata* Grube, 1860).

##### Material examined.

Alykes, Crete, Greece: CALB-15d-08 (1 ind.) [coll. 17.6.2008].

##### Type locality.

Balearic Islands (western Mediterranean Sea).

##### Distribution.

Pacific Ocean (Galápagos Islands), Red Sea, Mediterranean: WB, CB, AD, AS. New record for the Greek coast.

##### Habitat.

Shallow subtidal depths, on hard substrates, among photophilic algae, in *Posidonia oceanica* rhizomes and vermetid reefs.

#### Genus Trypanosyllis

Claparède 1864

##### Type species.

*Syllis zebra* Grube, 1860

#### Trypanosyllis coeliaca

Claparède, 1868

http://species-id.net/wiki/Trypanosyllis_coeliaca

Trypanosyllis coeliaca Claparède 1868: 513, pl. 13, fig. 3; Fauvel 1923: 270, figs 101f–h; Cognetti 1957: 27, fig. 5a; 1961: 296, Hartmann-Schröder 1979: 78; Perkins 1981: 1155, figs 33–34; Campoy 1982: 354; Uebelacker 1984: 93, fig. 88; San Martín 1984b: 274, fig. 63; 2003: 308, figs 169–170; Arvanitidis 1994: 109; Çinar 1999: 316, fig. 4.144; Çinar and Ergen 2003: 789.
Pseudosyllis brevipennis Grube, 1863: 44, pl. 4, fig. 5.

##### Material examined.

Haifa Bay, Israel, eastern Mediterranean Sea, Station ALA-IL-7 (1 ind.) [coll. 11.10.2009]. Alykes, Crete, Greece: CALA-10b-08 (1 ind.), CALB-10c-08 (1 ind.) [coll. 17.6.2008]; CALA-5a-08 (1 ind.), CALB-1d-08 (2 ind.), CALB-5a-08 (1 ind.) [coll. 18.6.2008]. Elounda, Crete, Greece: CELA-15b-07 (1 ind.), CELA-15c-07 (1 ind.) [coll. 26.9.2007]; CELB-5c-07 (1 ind.), CELA-10a-07 (1 ind.), CELB-10c-07 (1 ind.) [coll. 27.9.2007]; CELB-1a-07 (2 ind.), CELB-1e-07 (1 ind.) [coll. 29.9.2007]; CELB-10b-08 (1 ind.), CELA-15a-08 (1 ind.) [coll. 17.6.2008]; CELB-1b-08 (1 ind.), CELA-5b-08 (1 ind.), CELA-5c-08 (1 ind.), CELB-5c-08 (1 ind.), CELA-5d-08 (2 ind.) [coll. 18.6.2008].

##### Type locality.

Gulf of Naples (western Mediterranean Sea).

##### Distribution.

Circumtropical. Mediterranean Sea: WB, CB, AD, AS, LB. New record for the Israeli coast.

##### Habitat.

From infralitoral depths to 760 m, on hard substrates, among algae, corals, hydrozoans, sponges and *Posidonia oceanica* rhizomes, in vermetid reefs, in coarse sand.

##### Remarks.

Specimens from Greece have a faint or no visible trepan. Individuals without trepan but otherwise identical to *Trypanosyllis coeliaca* have in the past been identified as *Pseudosyllis brevipennis* Grube, 1863, but according to San Martín (2003) the absence of the trepan can be attributed to a number of reasons, including loss, and *Pseudosyllis brevipennis* is regarded as a synonym of *Trypanosyllis coeliaca*.

## Discussion

The present study yielded a number of species reported for the first time in the respective areas, and a high number of the new additions belong to the subfamily Exogoninae (Fig. 3). This could be explained by the fact that the small-sized individuals of this subfamily might have been overlooked in earlier works on the syllid fauna of the area which report only very few or no Exogoninae species at all (e.g. Fauvel 1957, Tebble 1959, Bellan 1964, Ergen 1976). The Exogoninae genus *Prosphaerosyllis*, which has recently been raised from subgeneric to generic level by San Martín (2005), has a difficult and confused taxonomy and several species have recently been described or transferred to the genus (Olivier et al. 2011, Çinar et al. 2011). Currently, 31 species of the genus are considered valid (including an unnamed one, see San Martín 2003), of which 11 have so far been reported to occur in the Mediterranean Sea (Table 3). However, several of the reported species in the area do in fact belong to other species, the identity of which can only be determined through thorough examination of the material in question. The presence of the Red Sea species *Prosphaerosyllis brevicirra* (Hartmann-Schröder, 1960) in the Mediterranean belongs to these doubtful records. Records of *Sphaerosyllis brevicirra* from the western Mediterranean Sea by Alós (1989) and from the Aegean Sea (Simboura 1996, Çinar 1999) belong to an undescribed *Prosphaerosyllis* species (San Martín 2003). These differ from *Prosphaerosyllis brevicirra* by the absence of dorsal cirri on chaetiger 2 (reported as present in Alós’ (1989) description but in fact absent (San Martín 2003)), the absence of the conspicious papilla on the dorsal cirrus and by thicker aciculae. Hartmann-Schröder (1960) does not mention the papilla on the dorsal cirrus in her description of the species (only visible in the illustrations, but confirmed through examination of type material); instead she focuses on the reduced length of the dorsal cirri as a character to distinguish the species from its congeners. This fact might have lead to confusion of *Prosphaerosyllis brevicirra* with other species possessing short dorsal cirri. Two other reports of the species from adjacent areas (Red Sea, Atlantic) likewise do probably not belong to *Prosphaerosyllis brevicirra*: Ben-Eliahu’s (1977a) redescription of the species based on material from the Gulf of Elat (Red Sea) differs in several aspects from Hartmann-Schröder’s (1960) description and from the type material. In particular, Ben-Eliahu does not mention or illustrate the papilla on the dorsal cirrus, her specimens have four eyes and one anterior pair of eyespots (eyespots, a character considered as invariable within species (Riser 1991), are absent in *Prosphaerosyllis brevicirra*) and the proventriculum occupies 4 chaetigers (3 in *Prosphaerosyllis brevicirra*). According to the description and illustrations, the species might in fact belong to *Prosphaerosyllis marmarae* (see remarks for this species above). The record of *Sphaerosyllis brevicirra* from the Spanish Atlantic coast (Parapar et al. 1994), though described as similar to Alós’ (1989) specimens, differs in fact from these by the presence of dorsal cirri on chaetiger 2 and much longer dorsal cirri. It also differs from *Prosphaerosyllis brevicirra* in having falcigers with serrated blades in anterior chaetigers, no papilla on the dorsal cirrus and much longer dorsal cirri anteriorly (140 µm vs ca. 20 µm in *Prosphaerosyllis brevicirra*). The species *Prosphaerosyllis brandhorsti* (Hartmann-Schröder, 1965) has been recorded in Italy by Gambi et al. (1995). However, the only other records of the species apart from its type locality (Isla Mocha, Chile) are from the northern Pacific (Banse 1972) and belong possibly to possibly *Prosphaerosyllis ranunculus* (Kudenov and Harris, 1995). The presence of *Prosphaerosyllis brandhorsti* in the Mediterranean Sea has thus to be considered as doubtful. An identification key to the currently valid Mediterranean species of *Prosphaerosyllis* can be found below.

**Table 3.** Reported distribution records of *Prosphaerosyllis* species in the Mediterranean. †= doubtful record, identity unknown. ‡= doubtful record, probably *Prosphaerosyllis* sp. [unnamed, San Martín 2003], §= doubtful record, probably *Prosphaerosyllis marmarae*. References: 1= this study, 2= San Martín 1984, 3= San Martín 2003, 4= Gambi et al. 1995, 5= Alós 1989, 6= Somaschini et al. 1994, 7= Lanera et al. 1990, 8= Zenetos et al. 1997, 9= Simboura 1996, 10= Çinar 1999, 11= San Martín et al. 1982, 12= Çinar and Ergen 2002, 13= Çinar et al. 2003, 14= Somaschini and San Martín 1994, 15= Çinar et al. 2011, 16= Katzmann, 1983, 17= Ben-Eliahu 1977a. Literature-based works (e.g. Musco and Giangrande 2005, Simboura and Nicolaidou 2001) are not included to avoid repetition of records.

**Species**	**Type locality**	**WB**	**AD**	**CB**	**AS**	**LB**
*Prosphaerosyllis adelae* San Martín, 1984	Balearic Islands, Spain, west Mediterranean	2, 3				1
*Prosphaerosyllis brandhorsti* (Hartmann-Schröder, 1965)	Isla Mocha, Chile, Pacific Ocean	4^†^				
*Prosphaerosyllis brevicirra* (Hartmann-Schröder, 1960)	Ghardaqa, Egypt, Red Sea	4^‡^, 5^‡^, 6^‡^, 7^‡^		8^‡^	9^‡^, 10^‡^	
*Prosphaerosyllis campoyi* (San Martín et al., 1982)	Andalusia, Spain, western Mediterranean	3, 11			1, 12	13
*Prosphaerosyllis chauseyensis* Olivier et al., 2011	Normandy, France, north-east Atlantic					1
*Prosphaerosyllis giandoi* (Somaschini and San Martín, 1994)	Tyrrenian Sea, Italy, western Mediterranean	14				
*Prosphaerosyllis longipapillata* (Hartmann-Schröder, 1979)	Broome, north-west Australia					3, 1
*Prosphaerosyllis marmarae* Çinar et al., 2011	Marmara Sea, Turkey, eastern Mediterranean				15	1
*Prosphaerosyllis* sp. [unnamed, San Martín 2003]	Cabo de Creus, Spain, western Mediterranean	3				
*Prosphaerosyllis tetralix* (Eliason, 1920)	Öresund, Sweden	3	16	8		17^§^
*Prosphaerosyllis xarifae* (Hartmann-Schröder, 1960)	Sarso, Egypt, Red Sea	3			12	

### Key to the Mediterranean species of *Prosphaerosyllis*

**Table:** 

1	Dorsal cirri on chaetiger 2 present	2
–	Dorsal cirri on chaetiger 2 absent	*Prosphaerosyllis* sp. [San Martín 2003]
2	Dorsal cirri and antennae with conspicuous papilla	*Prosphaerosyllis chauseyensis*
–	Dorsal cirri and antennae without conspicuous papilla	3
3	Papillae on dorsum arranged in regular longitudinal rows	4
–	Papillae on dorsum arranged irregularly	5
4	Pharynx through 4–5 chaetigers, pharyngeal tooth on midline of pharynx	*Prosphaerosyllis longipapillata*
–	Pharynx through 3 chaetigers, pharyngeal tooth in anterior third of pharynx	*Prosphaerosyllis tetralix*
5	Dorsal cirri papilliform	6
–	Dorsal cirri with bulbous cirrophore and rounded or elongated cirrostyle	7
6	Prostomium retracted under posterior chaetigers. Antennae and dorsal cirri distally truncated. Aciculae subdistally with a crown of spines	*Prosphaerosyllis adelae*
–	Prostomium not retracted under posterior chaetigers. Antennae and dorsal cirri distally rounded. Aciculae with subdistal swelling	*Prosphaerosyllis giandoi*
7	Palps densely papillated. Dorsal papillation inconspicious	*Prosphaerosyllis marmarae*
–	Palps with few or no papillae. Dorsum with distinct papillation	8
8	Blades of falcigers in midbody with strong serration	*Prosphaerosyllis campoyi*
–	Blades of falcigers finely serrated	*Prosphaerosyllis xarifae*

## Supplementary Material

XML Treatment for Syllides

XML Treatment for Syllides edentatus

XML Treatment for Syllides japonicus

XML Treatment for Myrianida

XML Treatment for Myrianida inermis

XML Treatment for Myrianida quindecimdentata

XML Treatment for Perkinsyllis

XML Treatment for Perkinsyllis augeneri

XML Treatment for Parapionosyllis

XML Treatment for Parapionosyllis elegans

XML Treatment for Prosphaerosyllis

XML Treatment for Prosphaerosyllis adelae

XML Treatment for Prosphaerosyllis campoyi

XML Treatment for Prosphaerosyllis chauseyensis

XML Treatment for Prosphaerosyllis longipapillata

XML Treatment for Prosphaerosyllis marmarae

XML Treatment for Prosphaerosyllis xarifae

XML Treatment for Salvatoria

XML Treatment for Salvatoria alvaradoi

XML Treatment for Salvatoria euritmica

XML Treatment for Salvatoria neapolitana

XML Treatment for Salvatoria vieitezi

XML Treatment for Salvatoria yraidae

XML Treatment for Sphaerosyllis

XML Treatment for Sphaerosyllis bulbosa

XML Treatment for Sphaerosyllis glandulata

XML Treatment for Sphaerosyllis gravinae

XML Treatment for Sphaerosyllis taylori

XML Treatment for Sphaerosyllis thomasi

XML Treatment for Opisthosyllis

XML Treatment for Opisthosyllis brunnea

XML Treatment for Syllis

XML Treatment for Syllis alternata

XML Treatment for Syllis compacta

XML Treatment for Syllis cruzi

XML Treatment for Syllis gerundensis

XML Treatment for Syllis jorgei

XML Treatment for Syllis pulvinata

XML Treatment for Syllis tyrrhena

XML Treatment for Syllis westheidei

XML Treatment for Trypanosyllis

XML Treatment for Trypanosyllis coeliaca

## Supplement information

The Scratchpads version of this publication is available at:

http://polychaetes.marbigen.org/node/1636

Figures are available at:

http://polychaetes.marbigen.org/category/image-galleries/eastern-mediterranean-syllidae

Locations and their description are available at:

http://polychaetes.marbigen.org/content/darwincorelocation

Tables with species occurences are available at:

http://polychaetes.marbigen.org/content/species-occurences-sampling-stations

http://polychaetes.marbigen.org/content/reported-distribution-records-prosphaerosyllis-species-mediterranean

The data underpinning the analysis reported in this paper are deposited at:

GBIF (Global Biodiversity Information Facility):

http://ipt.pensoft.net/ipt/resource.do?r=easternmedsyllids

Dryad Data Repository: doi: 10.5061/dryad.4b7k408g
